# Artificial intelligence (AI) in academic publishing: Legitimate use, plagiarism detection, and ethical challenges

**DOI:** 10.22038/ijbms.2026.27130

**Published:** 2026

**Authors:** Leila Arabi, Ali Roohbakhsh, Bizhan Malaekeh-Nikouei, Bibi Sedigheh Fazly Bazzaz

**Affiliations:** 1 Nanotechnology Research Center, Pharmaceutical Technology Institute, Mashhad University of Medical Sciences, Mashhad, Iran (Assistant Editor); 2 Pharmaceutical Research Center, Pharmaceutical Technology Institute, Mashhad University of Medical Sciences, Mashhad, Iran (Assistant Editor); 3 Biotechnology Research Center, Pharmaceutical Technology Institute, Mashhad University of Medical Sciences, Mashhad, Iran (Editor–in–Chief)

## Introduction

The rapid evolution of artificial intelligence (AI) continues to reshape the landscape of academic publishing. As machine learning and large language models (LLMs) become more sophisticated, their integration into scientific writing, peer review, and editorial processes promises to improve efficiency, accessibility, and research integrity. However, it also raises critical questions about authorship, authenticity, and ethical responsibility.

In this editorial of Iranian Journal of Basic Medical Sciences (IJBMS), we explore how AI can be legitimately used by authors and reviewers, how it assists in detecting plagiarism and data fabrication, and how emerging patterns of AI use among Generation Z researchers require new ethical awareness.

### Legitimate use of AI by authors

AI tools have become valuable aids for researchers seeking clarity, conciseness, and coherence in their manuscripts. Writing assistants such as ChatGPT or Grammarly can help improve grammar, style, and fluency, particularly for non-native English speakers. AI-based summarization tools can also extract large bodies of literature, helping authors identify relevant research gaps and trends (1). However, legitimate use requires transparency and accountability. According to the Committee on Publication Ethics (COPE) and several leading publishers, authors must disclose the use of AI tools and ensure that all intellectual contributions remain human. Importantly, AI-generated text should never be used without verification. As COPE emphasizes, authors are fully responsible for checking all factual accuracy, references, and potential bias introduced by automated systems (2). AI can support expression but not idea creation. Scientific reasoning, data interpretation, and hypothesis formulation must originate from human authors.

### AI in peer review: Potential and caution

Peer review is the foundation of academic quality control, yet it remains time-consuming, subjective and often inconsistent. AI offers promising support tools that can assist in pre-screening submissions for clarity, structure, or methodological completeness. Additionally, AI can help reviewers by suggesting relevant literature, verifying citation accuracy, or summarizing sections for faster evaluation. Nevertheless, AI should improve human reviewers. However, relying too much on algorithms may reinforce existing biases, particularly if the models were trained on unbalanced or outdated data (3). In addition, reviewers are advised against uploading any portion of a submitted manuscript into generative AI tools, as this action may compromise the authors’ confidentiality and proprietary rights. 

Therefore, journals should encourage a hybrid model of peer review, where AI performs mechanical checks, and human reviewers focus on conceptual depth and scientific integrity. Reviewers and editors must use AI tools only within secure, confidential frameworks and are prohibited from uploading manuscripts to external platforms that may compromise privacy or intellectual property.

### AI for plagiarism, data fabrication, and image manipulation detection

Perhaps the most impactful contribution of AI in academic publishing is in research integrity verification. Traditional plagiarism detection software is evolving into more advanced, AI-driven systems capable of identifying semantic or conceptual plagiarism (3).

AI also plays an increasing role in detecting data fabrication and image manipulation. Deep-learning models can analyze image metadata, detect duplicated microscopy figures, or notice unnatural statistical patterns. These tools have proven invaluable in large-scale editorial screening and even in post-publication investigations (4).

However, editors and reviewers must remember that AI tools provide signals, not a final judgement. Human judgment remains essential in determining whether an identified similarity or irregularity constitutes ethical misconduct. AI should therefore be seen as a guardian of integrity, supporting editors but never replacing editorial expertise and ethical judgment.

### Generation Z: New challenges and responsibilities

Generation Z researchers bring both innovation and risk to scholarly communication. Their comfort with AI-based writing and image-generation tools can accelerate research dissemination and creativity. However, it also introduces new ethical dilemmas.

Many young scholars may unintentionally cross ethical boundaries by relying too much on generative models for drafting, paraphrasing, or even producing figures without appropriate acknowledgment. In addition, some AI models fabricate fake references and citations and researchers should pay attention to this important issue. Therefore, journals and academic institutions must educate emerging researchers on the ethical boundaries of AI use. Workshops, author guidelines, and editorial statements could clearly define what constitutes legitimate versus unethical AI assistance.

### The IJBMS perspective and policy

As we mentioned in our previous editorial, IJBMS policy aligns with COPE’s position statement on the use of AI in manuscript preparation (5). We emphasized that authors are required to declare any AI tool used during writing, data analysis, or figure generation. 

AI can help authors refine language, assist reviewers in quality assurance, and enable editors to detect misconduct more effectively than ever before. We hope that employment of the AI-powered plagiarism and image-detection tools could uphold research integrity. We believe that when AI used responsibly, it enhances clarity, fairness, and precision in research dissemination. However, these benefits come with ethical boundaries. Transparency, disclosure, and accountability must guide every use of AI. In fact, Human creativity, critical thinking, and moral responsibility remain irreplaceable principles in science.

We wish our authors, reviewers, and readers a year of progress guided by both human and artificial intelligence, working together for the advancement of science.

## References

[1] Gao C, Howard F, Markov N, Dyer E, Ramesh S, Luo Y, Pearson A (2023). Comparing scientific abstracts generated by ChatGPT to real abstracts with detectors and blinded human reviewers. NPJ Digit Med.

[2] Arabi L, Roohbakhsh A, Malaekeh-Nikouei B, Fazly Bazzaz BS (2024). Joining COPE: Opportunities and benefits for the Iranian Journal of Basic Medical Sciences. Iran J Basic Med Sci.

[3] Giray L (2024). Benefits and challenges of using AI for peer review: A study on researchers’ perceptions. Ser Libr.

[4] Pellegrina D, Helmy M (2025). AI for scientific integrity: Detecting ethical breaches, errors, and misconduct in manuscripts. Front Artif Intell.

[5] Sadhin I, Hassan T, Nayim A (2023). Plagiarism detection using artificial intelligence. Int J Comput Inf Syst.

[6] Arabi L, Roohbakhsh A, Malaekeh-Nikouei B, Fazly Bazzaz BS (2025). The impact of artificial intelligence (AI) in academic writing and publication: Iranian Journal of Basic Medical Sciences (IJBMS) policy. Iran J Basic Med Sci.

